# The Dirty War Index: A Public Health and Human Rights Tool for Examining and Monitoring Armed Conflict Outcomes

**DOI:** 10.1371/journal.pmed.0050243

**Published:** 2008-12-16

**Authors:** Madelyn Hsiao-Rei Hicks, Michael Spagat

## Abstract

Madelyn Hsiao-Rei Hicks and Michael Spagat introduce the "Dirty War Index," a public health tool that identifies rates of undesirable or prohibited war outcomes inflicted on populations during armed conflict.

Documentation, analysis, and prevention of the harmful effects of armed conflict on populations are established public health priorities [1–5]. Although public health research on war is increasingly framed in human rights terms [6–13], general public health methods are typically applied without direct links to laws of war. Laws of war are international humanitarian laws and customary standards regarding the treatment of civilians and combatants, mainly described in the four Geneva Conventions of 1949 and their Additional Protocols I and II regarding international and civil conflicts [14]. With notable exceptions [11,15–17], absolute numbers are usually reported (e.g., number of persons killed), without systematic description of the proportional effects of armed conflict, thereby limiting the utility of findings and scope of interpretation.

In this paper, we introduce the “Dirty War Index” (DWI): a data-driven public health tool based on laws of war that systematically identifies rates of particularly undesirable or prohibited, i.e., “dirty,” war outcomes inflicted on populations during armed conflict (e.g., civilian death, child injury, or torture). DWIs are explicitly linked to international humanitarian law to make public health outcomes directly relevant to prevention, monitoring, and humanitarian intervention for the moderation of war's effects. After choosing the particular outcome to be measured, a DWI is calculated as:

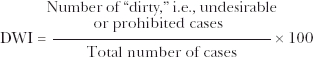

**Summary Points**

- War, a major public health problem, is a situation where the interests of public health, human rights, and humanitarian law intersect.
- The DWI is a data-driven public health tool that identifies rates of particularly undesirable or prohibited, i.e., “dirty,” outcomes inflicted on populations during war (e.g., civilian death, child injury, or torture).
- A DWI is calculated as: (Number of “dirty,” i.e., undesirable or prohibited cases/Total number of cases) × 100.
- DWIs are designed for direct, easy translation of war's public health outcomes into the human rights, policy, and interdisciplinary work needed to address war's practice.
- DWIs support monitoring, deterrence, and humanitarian intervention by explicit links to international humanitarian laws and by exposing rates of unacceptable combat outcomes (DWI values) from different weapons or combatant groups.

For example: In Table 1, we measure the DWI ratio of “Number of civilians killed/Total number of civilians and opponent combatants killed” using a casualty dataset for Colombia's civil conflict [18]. Table 2 links this DWI to relevant laws of war. DWI values of 99 for illegal paramilitaries, 46 for guerrillas, and 45 for government forces show that paramilitaries are “dirtiest” in terms of proportion of civilians constituting their victims of unopposed attacks (chi-square = 5,010, degree of freedom [df] = 2, *p* < 0.001). 99% of paramilitary victims were civilians and only 1% were military opponents. This finding, combined with the paramilitaries' methods (execution by close-range gunfire in massacres), suggests intentional targeting of civilians that requires recognition in Colombia's paramilitary demobilization, disarmament, and reintegration process [19].

**Table 1:**
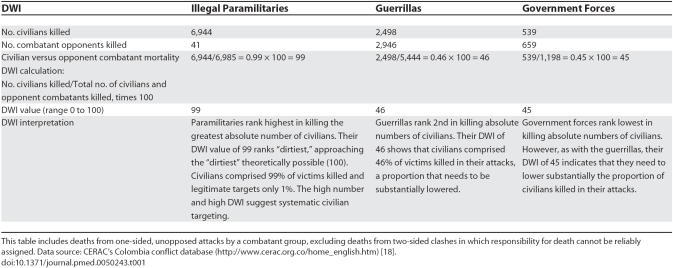
Dirty War Index for Attacks by Actors in the Colombian Civil Conflict, 1988–2005: Civilian Versus Opponent Combatant Mortality

**Table 2:**
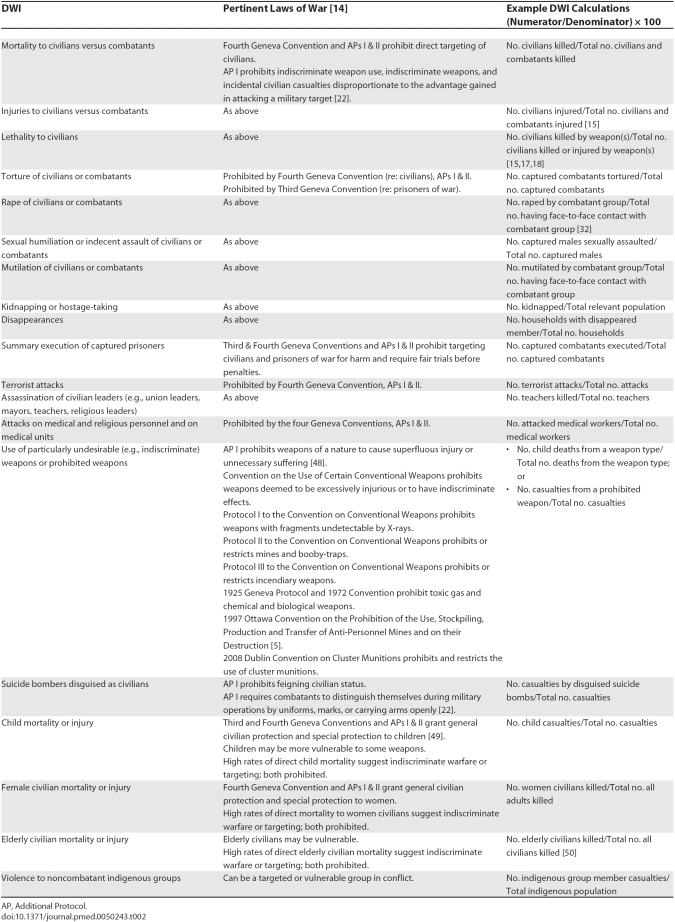
DWIs Suggested for Measuring Rates of Undesirable or Prohibited Outcomes from Aggression in Armed Conflict

As ratios, DWIs complement absolute numbers and lend themselves to comparisons over time, between wars, between weapons, and between warring combatant groups to identify better versus worse performers. Noncombatant wounded-to-killed ratios can provide evidence of war crimes [16]. Proportional “atrocity statistics” [20] from a Darfur survey substantiated US Secretary of State Colin Powell's declaration of genocide and the referral of Darfur's situation to the International Criminal Court [20,21]. By facilitating clear, systematic comparisons, DWIs can help analyze and expose how combatants engage in war and affect populations, thereby increasing the accountability of military and political leaders. This paper describes the theoretical basis and practical applications of the DWI, with brief examples from armed conflicts. More detailed DWI analyses of specific conflicts are planned for future papers.

## Calculating and Using DWIs

A DWI can be easily used and understood, facilitating interdisciplinary communication and research on war's effects. DWIs can measure rates of undesirable outcomes from accepted methods (e.g., civilian casualties from aerial bombing of military targets). They can also measure rates of using prohibited, illegitimate methods (e.g., torture), and rates of applying illegitimate methods to especially vulnerable populations (e.g., torturing children) to describe rates of exceptional atrocity. However, the mere application of DWI analysis to a combatant group does not indicate that it is “dirty”: a DWI ratio simply identifies how often, if at all, the group is linked with the particular undesirable outcome being measured, facilitating comparisons. To illustrate, we draw on data from B'Tselem (http://www.btselem.org/english/statistics/Index.asp), a nongovernmental organization that monitors casualties from the Israeli-Palestinian conflict. We apply a “female mortality DWI” (Number of females killed/Total number killed) to conflict-related killings from September 29, 2000 to April 30, 2007: Israeli security forces killed 213 females among 4,057 Palestinians (DWI = 5). Palestinians killed 283 females among 705 Israeli civilians (DWI = 40). Palestinians killed 10 females among 317 Palestinians (DWI = 3). Comparison of actors' DWIs shows significantly higher discrimination of female from male targets by Israeli security forces and by Palestinian actors when targeting Palestinians, and lower discrimination of female from male targets when Palestinian actors target Israeli civilians (chi-square = 833, df = 2, *p* < 0.001).

The best possible DWI value is 0, indicating that the objectionable outcome is identified in no measured cases. The worst possible DWI value is 100, indicating that the objectionable outcome is identified in 100% of measured cases. Any rate above 0 for prohibited actions or war crimes is unacceptable, and eliminating violations is imperative. DWIs for undesirable outcomes are less straightforward. The highly undesirable outcome of civilian harm is not prohibited by laws of war if combatants do everything feasible to distinguish between civilians and military targets (the principle of distinction), if they attempt to minimize incidental harm to civilians, and if they intend to avoid harming civilians in excess of anticipated military goals (the principle of proportionality) [1,22,23]. Civilian harm is also balanced against the “military necessity” of objectives [24]. Though what is feasible, proportional, or necessary is highly subjective [22–24], clearly the lowest possible rates should be sought for undesirable outcomes such as “incidental” civilian death. High DWI values for undesirable outcomes indicate extreme destruction, signal the need for close scrutiny, and may suggest war crimes.

Tables 2 and 3 list specific DWIs, their pertinent laws of war, and example calculations. Table 2 lists DWIs for undesirable or prohibited aggression in armed conflict. DWIs can be analyzed by demographic subgroup for indiscriminate warfare, disproportionate effects of targeting, or particular vulnerability to weapons. For example, with “casualties” defined as injuries or deaths, a “child casualty DWI” (Number of child casualties/ Total number of casualties) applied to weapons-casualty data from Chechnya [25] gives the following child casualty ratios for different explosive devices: antitank landmines (34/223, DWI = 15), antipersonnel landmines (223/1,004, DWI = 22), booby traps (65/214, DWI = 30), and other unexploded ordnance (UXO) (255/892, DWI = 29). DWIs indicate that in Chechnya, UXO and booby traps are more dangerous to children than landmines and significantly “dirtier” in this respect (chi-square = 25.0, df = 3, *p* < 0.001).

**Table 3:**
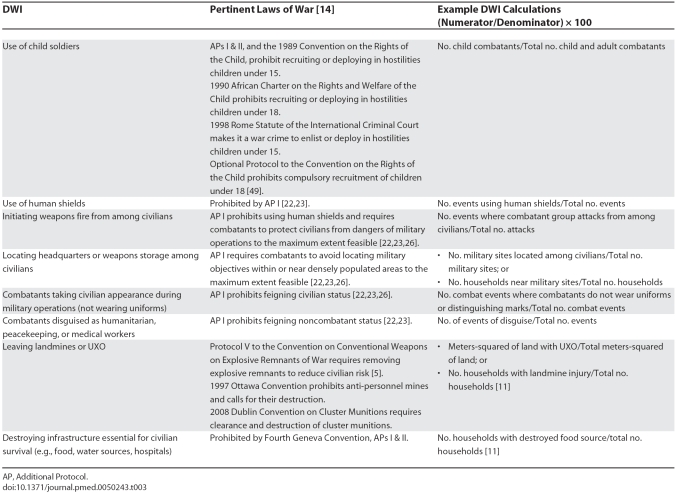
DWIs Suggested for Measuring Rates of Unacceptable Endangerment in Armed Conflict

Table 3 lists DWIs for unacceptable endangerment in armed conflict [14,23,24]. To illustrate, we apply the last DWI listed, “Destroying infrastructure essential for civilian survival (food, water, hospitals),” to survey data from eastern Burma where the Burmese military junta is in conflict with ethnic minority groups. The Burmese military regime destroyed or stole food from 472 of 1,813 surveyed households [11]. The Burmese military's DWI of 26 indicates a 26% rate of committing the humanitarian violation of destroying civilian food sources, associated in the study with significantly greater odds of household landmine injury (perhaps due to foraging for food), child malnutrition, and death [11].

In Table 4 we analyze the Northern Ireland conflict for two complementary DWIs: aggressive acts (killing civilians) and endangerment to civilians (by not wearing uniforms). Combatants who blur distinctions between themselves and civilians transfer their risk onto civilians [23,24]. Endangerment of noncombatants can be a byproduct of a method, as when guerrilla forces hide “among the people,” taking the battlefield to civilians [23,24,26]. Endangerment can also be a direct goal. As described by Viet Cong leaders [27] and American soldiers [23] in the Vietnam War, Viet Cong forces trained children to throw grenades at South Vietnamese and American soldiers, partly to provoke opponents to shoot children and bring shame to themselves and their force. Child soldiers are more often killed or injured than adult soldiers, being deployed at the front line, to lay or clear mines, or as suicide bombers because they provoke less suspicion [3,28,29]. To illustrate the issue of variable access to valid data for DWI applications, precise data for calculating child solider DWIs (Table 3) may be difficult to obtain for some conflicts. However, DWIs for using child soldier suicide bombers (Tables 2 and 3) could be highly accurate due to extensive media coverage of suicide attacks.

**Table 4:**
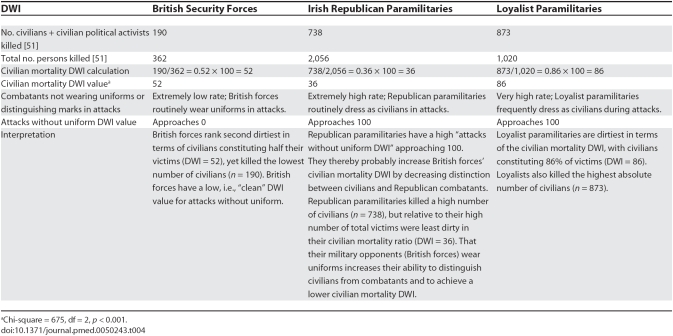
The Northern Ireland Conflict, 1969–2001: Complementary DWI Analyses for Unacceptable Aggression and Endangerment by Actors

DWI analysis can use any data source (media reports, epidemiological surveys, coroners' reports) as long as the data are adequately valid, accurate, and comprehensive. DWI analysis can be applied to event-based data or to aggregated data covering, for example, a year, a phase, or a whole conflict. Analysis of all DWIs supported by good data provides fuller description of a conflict and combatant behavior. A qualitative understanding of a conflict's nature and context is necessary for DWI application and interpretation. When possible, analysis should recognize when combatants avoid inflicting dirty outcomes, i.e., “clean” combat. DWIs suggest valuable data for prospective inclusion in conflict monitoring.

## Considerations

When DWIs are used to compare combatant groups or methods, it should not be assumed that those with the highest values are simply the dirtiest. Nor should it be assumed that lower DWI values “don't count.” A group may have a low DWI for recorded civilian mortality, but high DWIs for assassinating civilian leaders and disappearances. Another group may have low DWIs generally, and a very low DWI for torturing prisoners, but torture breaches the precepts of humanity utterly so that to have a measurable rate at all is deplorable.

DWIs reflect, in part, local conditions. For example, the lethality of civilian injuries reflects local treatment technology and access. It may therefore seem incorrect to compare DWIs for civilian lethality when health services differ. Similarly, it may seem unfair to compare child mortality DWIs between a conflict where children comprise a large proportion of the population and so are more likely to be killed and a conflict where children are few. However, researchers should not adjust for such factors when comparing DWIs across settings. This is because actors in armed conflict know, or are morally obliged to know, local resources and demographics and their implications for civilian harm. Combatants are obliged to take proportionately more care not to kill children when waging war in a child-dense population. Responsibility for dirty outcomes is not ameliorated by local conditions.

As for any conflict analysis [2,3,30], DWI selection, application, and interpretation must recognize the potential, varied biases of data sources and of particular DWIs. Conflicts are highly politicized, and combatants, supporters, and detractors have always tried to manipulate reports of war outcomes. Combatants may attempt to construct more favorable DWIs not only by decreasing dirty combat, but by concealing dirty outcomes, or by misrepresenting or provoking opponents' dirty outcomes. For example, a group might attempt to raise an opponent's child mortality DWI by using child soldiers or children as human shields.

Some DWI outcomes, such as injuries, may tend to be under-reported [16]. War-associated rape may be difficult to measure due to stigma and under-reporting, though substantial reports exist [21,24,31,32]. Although bias can affect DWI values, as ratios DWIs are relatively less affected by under- or over-counting than absolute numbers. For example, if a population generally under-reports war-related rape by 40%, this does not bias comparing rates between different combatant groups.

DWIs, complemented by absolute numbers, can suggest strategic aspects of actors' methods. For example, systematic civilian targeting is suggested by combined findings of: many events killing or injuring civilians; high ratios of civilian versus combatant mortality; frequent use of methods causing high civilian casualties; frequent use of methods causing high civilian lethality; and high rates of civilian harm from methods that are inherently “targeted” (handguns, machetes). Such proportional and numerical findings on civilian casualties have been used as evidence in International Criminal Court trials to establish systematic patterns indicating war crimes [33].

## DWIs Measure Outcomes, Not Justifications or Intentions

DWIs focus on whether the practice of war is just (*jus in bello*) and ignore whether the reason for war is just (*jus ad bellum*), separating two logically distinct moral issues in war [23]. We focus on practical outcomes because justifications for war are contested, are used to legitimize dirty combat, and can bias examination of war's impact [24,27,34–36]. Combatants and their supporters may believe or describe methods as just, whether the method is suicide bombing [24,37,38] or the World War II targeting of civilians by Germany and by the Allies with carpet bombing, fire-bombing, and atomic bombs directed at cities [23,24,26,27].

Although intentions affect combat outcomes, such as civilian mortality rates [16,27,34,35], we separate DWIs from intentionality for the following reasons. Intentions are contested, obscured, and distorted [3,35]. Dirty outcomes can result from malicious intent, beneficent intent, or recklessness (lack of intent to take due care). Frequently, combatant violence that appears wanton, sadistic, or vengeful (e.g., rape, mutilation) is mobilized by political actors for hidden strategic aims [24,27,35,39,40]. Combatants' intended effects may be disrupted by targets or adversaries [41]. Individual combatant behavior reflects overriding goals and sociocultural aspects of larger groups [34,35,38].

Accommodating intentions or justifications in DWIs would imply that good intentions or a “just war” attenuate responsibility for bad outcomes; an implication that is morally and legally refuted [36]. DWIs therefore only recognize the crucial matter of outcomes: the killing, injury, or abuse of individuals and populations who should be protected from war.

## Potential Deterrent Effect of the Dirty War Index

We choose the term “Dirty War Index” for three reasons. First, it unites moral, humanitarian, and scientific values inherent to most armed conflict research. Second, it avoids euphemisms that sanitize descriptions of war-induced public harm [12,13,27,42]. Third, emotional and cultural implications of “dirty” versus “clean” may heighten the sensitivity of combatant groups to the index, increasing its potential deterrent effect. No nation or combatant group wants to be considered “dirty” or described as dirtier than others.

Increased accountability can have a deterrent effect in armed conflict and encourages adherence to international humanitarian law; an important element in preventing violence towards noncombatants [1,24]. DWIs increase scrutiny and accountability specifically for dirty war methods. DWIs are analogous to corruption and bribery indices used by nongovernmental organizations and the World Bank to improve international governance through public monitoring and ranking governments by corruption [43,44]. In *Better: A Surgeon's Notes on Performance* [45], Atul Gawande describes how systematic analysis of war casualties reveals problems and suggests solutions, and how identifying exemplary performers can improve general performance. The DWI is developed for systematic, data-driven identification of relatively good versus bad performance, heightening its potential to stimulate positive change.

Military and political leaders not only want to win wars. They also seek superior moral authority [23]. Moral authority has social currency, creating better access to material resources, support, and security within local and international communities. To improve behavior in combatants and politicians insufficiently motivated by altruism, harnessing such self-interest is crucial. Exposure of atrocities through DWIs can put reputation, legitimacy, future resources, threat of retaliation, or power itself at stake [24].

As comparative rates, DWIs evoke the potential for change. The possibility of becoming “cleaner” may appeal to some offenders [24]. Actors may compete for better outcomes relative to military opponents, relative to in-group political competitors, or relative to themselves over time. A DWI's potency can be increased by engagement with social, cultural, and religious values of actors and their communities: honor versus dishonor [24], gaining versus losing “face,” shame versus pride, dignity versus humiliation [37,46], sacred versus profane [37], and valuing mercy and the lives of innocents [47]. Terms other than “Dirty War Index,” e.g., the “Dishonorable War Index,” could be used to greater effect in different contexts.

War and its destruction trigger emotions and self-interests that can obscure analysis by threatening us so that we revert to familiar prejudices, reactions, and cognitive frameworks. Through a public health approach using valid, precise proportional rates as outcomes, DWIs can help us and our audiences to detach from political biases and break through psychological denial when considering actors or methods in war. DWIs can present conflict data from a new perspective, thereby encouraging actors in war to reassess their combat methods, accountability, and interests.

**Linked Perspectives**

This Policy Forum is further discussed in two *PLoS Medicine* Perspectives:

Taback N (2008) The Dirty War Index: Statistical issues, feasibility, and interpretation. PLoS Med 5(12): e248. doi:10.1371/journal.pmed.0050248

Sondorp E (2008) A new tool for measuring the brutality of war. PLoS Med 5(12): e249. doi:10.1371/journal.pmed.0050249

## Abbreviations

df: degree of freedom
DWI: Dirty War Index
UXO: unexploded ordnance
